# Influence of cow and heifer categories on the hematology of Murrah buffaloes (*Bubalus bubalis*) raised in the microregion of Santarém, Pará, Brazil

**DOI:** 10.29374/2527-2179.bjvm003025

**Published:** 2025-10-07

**Authors:** Thiago Nogueira da Silva, Rubens Lima de Andrade, Carlos Eduardo Lima Sousa, Katarina Cardoso de Carvalho, Lael Evaristo Alves, Douglas Ferreira de Moraes, Raimundo Nonato Colares Carmago-Junior, Cláudio Vieira de Araújo, Kedson Alessandri Lobo Neves, Edmilson Cássio Braga Nogueira, Eldinei Mesquita Mendes, Welligton Conceição da Silva

**Affiliations:** 1 Centro Universitário da Amazônia. Santarém, PA, Brazil.; 2 Departamento de Medicina Veterinária, Instituto Federal de Educação, Ciência e Tecnologia do Pará, Santarém, PA, Brazil.; 3 Universidade Federal do Mato Grosso (UFMT), Sinop, MT, Brazil.; 4 Universidade Federal do Oeste do Pará (UFOPA), Santarém, PA, Brazil.

**Keywords:** blood cell count, hematocrit, leukocytes, platelets, hemograma, hematócrito, leucócitos, plaquetas

## Abstract

The hematological profile is indicated to evaluate the physiological condition of buffaloes, providing information on the functionality of production systems and their response to environmental factors. In tropical regions, such as the Lower Amazon, high temperature and relative humidity can lead to heat stress, affecting hematological parameters. Therefore, the objective of this study was to evaluate the influence of the cow and heifer categories on the hematology of female Murrah water buffaloes (*Bubalus bubalis*) raised in the Microregion of Santarém, Pará, Brazil. Blood samples were collected from 90 Murrah buffalo females, divided into two categories: heifers (22 to 24 months) and cows (36 to 38 months). A complete blood count was performed, considering the red and white series. The T test was applied to compare the means of parameters between categories (heifers and cows), using the RStudio Desktop 1.3.1093 software. There was a difference in hemoglobin (*p* = 0.0424), hematocrit (*p* = 0.0090) and MCV (*p* = 0.0393) higher in heifers and MCHC (*p* = 0.0289) in cows. On the other hand, there was no difference for the parameters red blood cells (*p* = 0.4363), MCH (*p* = 0.1042) and RBC (*p* = 0.6722). In relation to the white series, there was a difference in the absolute values of leukocytes (*p* = 0.0069), lymphocytes (*p* = 0.0008), atypical lymphocytes (*p* = 0.0351) and monocytes (*p* = 0.0477), being higher in heifers than in cows. On the other hand, the segmented ones (*p* = 0.0002) were higher in cows when compared to heifers. The other leukocyte parameters did not present statistical difference (*p* > 0.05). Therefore, there were higher amounts of white and red cells for heifers when compared to cows.

## Resumo

O perfil hematológico é indicado para avaliar a condição fisiológica de búfalos, fornecendo informações sobre a funcionalidade dos sistemas de produção e sua resposta a fatores ambientais. Em regiões tropicais, como o Baixo Amazonas, a alta temperatura e a umidade relativa podem levar ao estresse térmico, afetando parâmetros hematológicos. Portanto, o objetivo deste estudo foi avaliar a influência das categorias vaca e novilha sobre a hematologia de búfalas Murrah (*Bubalus bubalis*) criadas na Microrregião de Santarém, Pará, Brasil. Foram coletadas amostras de sangue de 90 fêmeas Murrah, divididas em duas categorias: novilhas (22 a 24 meses) e vacas (36 a 38 meses). Foi realizado hemograma completo, considerando as séries vermelha e branca. O teste *t* foi aplicado para comparar as médias dos parâmetros entre as categorias (novilhas e vacas), utilizando o software RStudio Desktop 1.3.1093. Houve diferença nos valores de hemoglobina (*p* = 0,0424), hematócrito (*p* = 0,0090) e VCM (*p* = 0,0393) mais elevados em novilhas e CHCM (*p* = 0,0289) mais elevado em vacas. Por outro lado, não houve diferença para os parâmetros hemácias (*p* = 0,4363), HCM (*p* = 0,1042) e RBC (*p* = 0,6722). Em relação à série branca, houve diferença nos valores absolutos de leucócitos (*p* = 0,0069), linfócitos (*p* = 0,0008), linfócitos atípicos (*p* = 0,0351) e monócitos (*p* = 0,0477), sendo maiores em novilhas do que em vacas. Por outro lado, os segmentados (*p* = 0,0002) foram mais elevados em vacas quando comparadas às novilhas. Os demais parâmetros leucocitários não apresentaram diferença estatística (*p* > 0,05). Portanto, houve maiores quantidades de células brancas e vermelhas nas novilhas em comparação às vacas.

## Introduction

Buffalo farming in Brazil has established the economy through strategic livestock activity, standing out for raising buffaloes for the production of high-quality meat and milk (Li et al., 2019; Khan et al., 2022; Silva et al., 2021). In addition, the growth in buffalo farming provides an increase in the country's economy, promoting the valorization of flooded areas, used by the animals for thermoregulation (Marquesini & Titto, 2023; Silva et al., 2021).

The physiological characteristics of these animals suffer significant climatic influences, directly affecting their metabolism, growth, reproduction, and immune response (Silva et al., 2023; Trapanese et al., 2024). In tropical regions, such as the Lower Amazon, high temperature and humidity can lead to heat stress, compromising feed intake and herd productivity (Silva et al., 2024a). However, rustic species, such as buffaloes, have physiological adaptations that favor survival in adverse environments, such as thermoregulation and fibrous forage digestion (Gerude Neto et al., 2023).

The hematological profile is indicated to assess the physiological condition of these animals, providing information on the functionality of production systems and their response to environmental factors (Chen & Luo, 2023). Hematological parameters, such as erythrocytes, hemoglobin, hematocrit, and leukocytes, reflect the body's ability to maintain homeostatic balance in the face of nutritional, infectious, and environmental challenges (Shah et al., 2024; Toro et al., 2023).

In the Lower Amazon, climatic and nutritional conditions can influence the blood composition of buffaloes (Rodrigues et al., 2024). Variations in air temperature, relative humidity and pasture quality directly impact the physiology of the animals, making it necessary to periodically evaluate hematological parameters. In addition, understanding the differences between heifers and adult cows helps to establish specific nutritional and sanitary protocols for each age group, ensuring efficient growth and production (Aquino et al., 2024; Islam et al., 2019; Silva et al., 2024b; Ventura et al., 2021).

The investigation of these parameters will allow a better understanding of the physiological conditions of these animals and will assist in the formulation of management strategies that promote the health and productivity of the buffalo herd in the Lower Amazon region. Therefore, the objective of this study was to evaluate the influence of the category on the hematology of female Murrah water buffaloes (*Bubalus bubalis*) raised in the Microregion of Santarém, Pará, Brazil.

## Material and methods

### Ethical aspects

The study was submitted to the Ethics Committee on the Use of Animals (CEUA) and was approved, with protocol number 0520230262 by the Federal University of Western Pará (UFOPA).

### Experimental area and climate

This study was developed in the micro-region of Santarém, Pará, in detail in the municipalities of Santarém, Alenquer and Mojuí dos Campos. The climate of the region is hot and humid (Am4), according to the classification proposed by Köppen and Geiger (1928), with annual precipitation between 1,900 and 2,100 mm, average annual temperature of 25.6^0^C, and relative humidity with variability from 84 to 86%, with the wettest quarter located between the months of February and April and the least rainy, between August and October (Martorano et al., 2017).

### Animals and management

A total of 90 Murrah buffalo females, non-lactating, non-pregnant, non-neutered, and clinically healthy, with a body condition score equivalent to three (scale of 0 to 5 - Silva et al., 2022), were used. Females were divided into two categories: heifers (*n* = 45) aged between 22 and 24 months, with an average weight of 430±45.2 kg and cows (*n* = 45) aged between 36 and 38 months, with an average weight of 670±52.5 kg. Females were vaccinated against foot-and-mouth disease and brucellosis, as well as dewormed three times a year.

The collections were carried out in April, at 6:30 am, the quietest period of the day in the region, equivalent to the rainiest period of the year. The females were managed in an extensive rearing system, in *Brachiaria brizantha* cv. Marandú, with water and salt *ad libitum* in all the properties visited. The animals were led slowly and calmly to the trunk so that the collections could be carried out, avoiding stress.

### Collection, processing and analysis of samples

Antisepsis was performed with 70% alcohol in the tail targeting the caudal vein, collecting a volume of 5 mL of blood. The blood was stored in 5 mL tubes with anticoagulant (Ethylenediamine Tetraacetic Acid – EDTA) and stored in Styrofoam containing gelox and then taken to the outsourced laboratory responsible for the analyses.

Hematological analyses were performed using the BC-2800Vet® automatic counter (Shenzhen Mindray Bio-Medical Electronics®, Germany) calibrated for the buffalo species.

Hemoglobin (g/dL) was determined and hematocrit (He-%), confirmed by capillary centrifugation, the hematimetric indices mean corpuscular volume (MCV - %), mean corpuscular hemoglobin concentration (MCHC; g dL^−1^ - %), mean corpuscular hemoglobin (MCH; pg), erythrocyte coefficient of variation (RBC; M μL^−1^) and platelet-derived indices (mean platelet volume – MPV), platelet size distribution amplitude index (PDW) and platelet count (PCT) were calculated.

The BC-2800Vet® counts red blood cells (x10^3^/uL), absolute and relative leukocytes (μL) (%) (relative segmented pSGM, SEGM = Absolute segmented, EOSP = Relative eosinophils, EOS = Absolute eosinophils, LINFp = Relative lymphocytes, LINF = Absolute lymphocytes, LINATp = Relative atypical lymphocytes (large preemies), LINAT = Absolute atypical lymphocytes, MONp = Relative monocytes, MON = Absolute monocytes).All results were evaluated by professionals in the field, reviewing slides under a microscope and the observed parameters..

The differential leukocyte count was performed by counting 100 cells in blood smears stained by the Diff-Quick (NEWPROV Produtos para Laboratório Ltda, Pinhais/PR). The analyses were carried out using the methodology described by Fontes et al. (2014), adapting it for buffaloes.

### Statistical analysis

All variables were tested for normality of residuals using the Shapiro-Wilk test and for homogeneity of variances using the Levene test. For each hematological parameter evaluated, the confidence intervals (CI) of the mean were estimated with a 95% probability. The calculation was carried out using Student's t-distribution, which is suitable for small sample sizes and unknown variances.

For the statistical analysis comparing the means between heifers and cows for each parameter assessed, the two-sample t-test was applied. Initially, the F-test was carried out to verify the homogeneity of the variances between the groups, considering the hypotheses H_0_: σ_1_^2^ = σ_2_^2^ (equal variances) and H_1_: σ_1_^2^ ≠ σ_2_^2^ (different variances), where sub-indices 1 and 2 correspond to the highest and lowest variance estimates, respectively.

The procedure adopted for the t-test was defined according to the result of the F-test. If the variances were homogeneous (acceptance of H_0_), the t-test was used with equal variances, adopting the joint variance. On the other hand, in the event of heterogeneity of variances (rejection of H_0_), the t-test was applied for unequal variances, according to the expression. In both cases of the t-test, the null hypothesis tested (H_0_) is equal to H_0: X ¯=Y ¯, against H_1: (X≠) ¯Y ¯, in all cases a significance level of 0.05 was adopted, using the Statistical Analysis System (SAS) On Demand for Academics software (SAS Institute Inc., 2024).

## Results

Table 1 shows the erythrocyte patterns of heifers and Murrah cows managed in the microregion of Santarém, Pará.

**Table 1:** Estimates of Average, standard deviations (SD), and lower (LB) and Upper (UL) limits of the 95% confidence interval for erythrocyte parameters in heifers and buffalo cows raised in the microregion of Santarém, Pará.

	Heifers		Cows		*p - value*
Parameters	**Average**	**CI**	**Average**	**CI**	
Red blood cells (×10^3^/μL)	5.91±1.99	(5.08-6.73)	6.09±1.29	(5.70-6.48)	0.4399
Hemoglobin (g/dL)	11.94±1.68	(11.24-12.63)	10.93±1.61	(10.45-11.42)	0.0430
Hematocrit (%)	34.85±5.99	(29.08-32.38)	30.73±5.49	(29.08-32.38)	0.0092
MCV (fl)	52.42±14.2	(48.15-56.68)	52.42±14.2	(48.15-56.68)	0.0399
MCH (pg)	18.54±4.09	(17.31-19.77)	18.54±4.09	(17.31-19.77)	0.1055
MCHC (%)	35.80±2.44	(35.07-36.54)	35.80±2.44	(35.07-36.54)	0.0293
RBC (%)	17.75±1.21	(17.38-18.11)	17.75±1.21	(17.38-18.11)	0.6766

Note: MCV = Mean Corpuscular Volume, MCH = Mean Corpuscular Hemoglobin, MCHC = Mean Corpuscular Hemoglobin Concentration, RBC = Red Blood Cell. CI = Confidence interval.

Table 2 shows the leukocyte patterns of buffalo heifers and cows managed in the micro-region of Santarém, Pará.

**Table 2:** Estimates of Average, standard deviations (SD), and lower (LB) and Upper (UL) limits of the 95% confidence interval of leukocyte and platelets parameters (analyzed in the automated hematological counter or counted using specific leukometry by microscopy) observed in buffalo heifers raised in the microregion of Santarém, Pará.

Parameters	Heifers		Cows		*p - value*
	Average	CI	Average	CI	
Leukocytes (x1000/μL)	16.953±7.42	(5.634-14.271)	9.742±2.759	(8.913-10.571)	0.0070
BASTP (%)	0.16±0.80	(-0.17-0.49)	0.04±0.20	(-0.01-0.10)	0.2981
BAST (µL)	0	-	3.55±16.75	(-1.47-8.59)	0.2981
SEGMp (%)	52.20±8.86	(48.54-55.85)	61.04±8.66	(58.44-63.64)	0.0002
SEGM (μL)	8.634±13.159	(3.202-14.066)	5.840±1.397	(5.420-6.260)	0.2393
EOSp (%)	0.88±1.06	(0.39-1.28)	0.62±0.90	(0.34-0.89)	0.4173
EOS (µL)	97.88±142.84	(38.91-156.84)	50.75±74.03	(28.51-72.99)	0.2878
LINFp (%)	39.36±8.45	(35.86-42.85)	30.84±9.13	(28.10-30.58)	0.0003
LINF (μL)	6.959±12.477	(1.808-12.109)	3.102±1.530	(2.642-3.562)	0.0008
LINATp (%)	0.28±0.61	(0.02-0.53)	0.06±0.33	(-0.03-0.16)	0.0416
LINATE (μL)	30.04±68.87	(1.61-58.46)	4.91±24.11	(-2.33-12.15)	0.0361
MONp (%)	7.60±2.66	(6.50-8.59)	7.34±2.67	(6.57-8.18)	0.8862
MON (μL)	1.259±1.928	(4.635-2.055)	7.397±396.02	(620.77-8.587)	0.0484
PLAQ (μL)	154.214±577.56	(151.525-210.866)	147.933±54.588	(128.528-227.337)	0.0872

Note: BASTp = Relative rods; BAST = Absolute rods; SEGMp Segmented relative; SEGM = Absolute segmented; EOSp = Relative eosinophils; EOS = Absolute eosinophils; LINFp = Relative lymphocytes; LINF = Absolute lymphocytes; LINATp = Relative Atypical Lymphocytes; LINAT = Absolute atypical lymphocytes; MONp = Relative monocytes; MON = Absolute monocytes; PLAQ = Platelets. CI = Confidence interval.

There was a difference in hemoglobin (*p* = 0.0424), hematocrit (*p* = 0.0090), and MCV (*p* = 0.0393), being higher in heifers when compared to cows. On the other hand, the MCHC (*p* = 0.0289) was higher than the number of cows. There was no difference for the red blood cells (*p* = 0.4363), MCH (*p* = 0.1042) and RBC (*p* = 0.6722) parameters.

There was a difference in the absolute values of leukocytes (*p* = 0.0069), lymphocytes (*p* = 0.0008), atypical lymphocytes (*p* = 0.0351) and monocytes (*p* = 0.0477), being higher in heifers. On the other hand, the segmented (*p* = 0.0002) were higher in cows. The other leukocyte parameters did not show statistical difference.

## Discussion

In this study, it was possible to determine the lower and upper values for the erythrocyte and leukocyte series of female Murrah buffaloes in different categories in the Microregion of Santarém, Pará, which can be used as reference values for future studies in the region. In addition, it was possible to present the comparison between the two categories of buffaloes describing differences between them.

With the improvement of laboratory techniques in veterinary clinics and the advancement of hematology, it has been evidenced in the literature that aspects of environmental conditions, type of breeding, feeding, feed, sex, age (Jaramillo et al., 2024) and edaphoclimatic aspects, interfere with blood components in several species (Jain, 1993) and these conditions lead to changes in the normal patterns described by several researchers (Martini et al., 2019; Paján-Jiménez et al., 2023). In addition to species-specific characteristics, physiological adaptations can influence blood components and the interpretation of blood count results (Thrall, 2015).

In this context, the analysis of hematological parameters between heifers and buffalo cows revealed differences in hemoglobin, hematocrit, MCV and MCHC values. The higher concentrations of hemoglobin and MCV observed in heifers are related to the increasing physiological and metabolic rate associated with intense hematopoiesis (Patel et al., 2016), which can help in the transport of oxygen due to higher energy demand. The MCHC was higher in cows, suggesting changes in erythrocyte morphology and hemoglobin concentration according to the period of animal development (Enculescu et al., 2020).

In another analysis carried out with buffaloes, samples from 73 Murrah animals were examined, divided into three groups. The results revealed the influence of age on the blood count values. The overall count of red blood cells, MCV, hemoglobin, platelets, leukocytes, segmented neutrophils, and lymphocytes were higher in young animals compared to adults (Fontes et al., 2014), results similar to those of the present study.

There was a difference in leukocyte patterns with higher values in heifers of leukocytes, lymphocytes, atypical lymphocytes and monocytes. The increase in the total amount of leukocytes and lymphocytes is due to the discrepancy in the immune response between heifers and cows (Farschtschi et al., 2022; Joo et al., 2021). The immune system of heifers tends to be more active, as the body is developing, which points to an increase in the production and circulation of lymphocytes (Fabjanowska et al., 2023; Horst et al., 2021). Exposure to new environments or pathogens stimulates atypical lymphocytes to be present in the circulation, which is normal in the growth phase (Earley et al., 2023).

The alteration in monocytes also points to an alternation in the inflammatory or immunological response between these groups. Thus, due to the physiological adaptation of heifers, monocytes tend to increase, as they are fundamental cells in the body's line of defense, while in cows the immune system is more balanced and does not activate this mechanism constantly (Rainard et al., 2022; Sorokina et al., 2022).

Rocha et al. (2021) observed that animals up to 11 months of age manifested a higher number of monocytes when compared to buffaloes aged 12 to 23 months, gradually decreasing with age. The authors justified that erythrocyte replacement is essential in younger individuals, requiring greater phagocytosis in the phagocytic monocytic system, present in organs such as the spleen, liver, and bone marrow. The macrophages, responsible for this extravascular hemolysis, correspond to the mature cells of the monocytic lineage, when they migrate to the tissues. This fact justifies the findings of the present study.

Regarding the relative values, the change in the amount of segmented neutrophils may indicate differences in the performance of the innate and acquired immune response (Fiorenza et al., 2021; Pascottini et al., 2021). The greater amount of neutrophils in cows may be related to the reproductive cycle of females, making them more susceptible to invading agents (Amin et al., 2023; Wrenzycki, 2022). The other leukocyte parameters do not indicate statistical variations, which suggests that some components of the immune response are similar between the groups, preserving a physiological homeostasis in the buffalo species (Ciliberti et al., 2025; Scatà et al., 2024).

The animals came from different livestock properties, and even though they received the same type of food, water, salt, the environment may have intricate characteristics that can be adopted as strategic points for future studies.

## Conclusion

The lower and upper limit values of Murrah heifers and cows raised in the Microregion of Santarém were determined. Higher levels of white and red cells were observed for heifers. The highest indices in the red series were hemoglobin and MCV for heifers and MCHC in cows. For the white series, there was a difference between leukocytes, lymphocytes and atypical lymphocytes, as well as monocytes, which were higher in heifers, which may be explained by the fact that they are young animals with more active immune responses to stimuli from the environment.
